# Histamine poisoning and cheese

**DOI:** 10.1186/2045-7022-5-S3-P107

**Published:** 2015-03-30

**Authors:** Chrys Tsakona

**Affiliations:** 1 Immunology Department, Dudley Group of Hospitals NHS Foundation Trust, Dudley, UK

## 

Scombrotoxin Poisoning is an atypical food poisoning closely resembling an allergic reaction. It is caused by release of (mainly) histamine into the flesh of freshly caught fish, prawns and blue or Swiss cheeses left for a while in room temperature. The typical symptoms are a peppery taste, generalised erythema and burning, facial swelling, headache, dizziness, fainting, sweating, numbness of the limbs which sometimes feel (intermittently) completely paralysed, and less often abdominal pain with violent vomiting and diarrhoea and rarely retrosternal tightness and pain, pulmonary oedema and coronary syndrome. As the diagnosis is clinical, based only on the symptoms, it is often misdiagnosed as allergy or food poisoning and a big number of cases is missed and unrecorded. A simple official assay developed by FDA to quantitate histamine it is not realistically usable diagnostically as histamine is a very short-lived molecule. Scombrotoxin poisoning was first described with fish with high levels of free histidine, like scombroid, and it is still associated with fish, particularly tuna. And although now there is some awareness about the risks associated with amateur fishing and Public Health Authorities have enforced safety measures, scombrotoxin poisoning from cheese is completely overlooked and reports are a single digit figure worldwide.

We present a 25 year old female who had a ham and cheese crepe in her honeymoon in Paris and within seconds she felt very unwell with all the above. She was given adrenaline with no prompt response of the ingredients buckwheat flour was blamed but the result was equivocal whereas the whole clinical picture was typical scombrotoxin poisoning rather than an allergic reaction and the patient has been advised accordingly.

It is essential that the clinicians are aware of this potentially life-threatening problem and the public is advised not to leave cheese for long out of the fridge. The high susceptibility of certain individuals (not all people who will eat from the same food will have a reaction) is genetically determined and after they experience a reaction they should execrcise extreme caution.

## Consent

Written informed consent was obtained from the patient for publication of this abstract and any accompanying images. A copy of the written consent is available for review by the Editor of this journal.

